# The obesity epidemic and rising diabetes incidence in a low-income racially diverse southern US cohort

**DOI:** 10.1371/journal.pone.0190993

**Published:** 2018-01-11

**Authors:** Baqiyyah N. Conway, Xijing Han, Heather M. Munro, Amy L. Gross, Xiao-Ou Shu, Margaret K. Hargreaves, Wei Zheng, Alvin C. Powers, William J. Blot

**Affiliations:** 1 Department of Epidemiology and Biostatistics, University of Texas Health Science Center, Tyler, Texas, United States of America; 2 International Epidemiology Field Station, Vanderbilt Institute for Clinical and Translational Research, Vanderbilt University Medical Center, Rockville, Maryland, United States of America; 3 Division of Epidemiology, Department of Medicine, Vanderbilt University School of Medicine, Nashville, Tennessee, United States of America; 4 Department of Internal Medicine, Meharry Medical College, Nashville, Tennessee, United States of America; 5 Division of Diabetes, Endocrinology and Metabolism, Vanderbilt University School of Medicine, Nashville, Tennessee, United States of America; East Tennessee State University, UNITED STATES

## Abstract

**Background:**

Obesity is known to be a major risk factor for diabetes, but the magnitude of risk and variation between blacks and whites are less well documented in populations heavily affected by obesity. Herein we assess rates and risks of incident diabetes in a diverse southern population where obesity is common.

**Methods:**

A total of 24,000 black and 14,064 white adults aged 40–79 in the Southern Community Cohort Study with no self-reported diabetes at study enrollment during 2002–2009 was followed for up to 10 (median 4.5) years. Incidence rates, odds ratios (OR) and accompanying 95% confidence intervals (CI) for medication-treated incident diabetes were determined according to body mass index (BMI) and other characteristics, including tobacco and alcohol consumption, healthy eating and physical activity indices, and socioeconomic status (SES).

**Results:**

Risk of incident diabetes rose monotonically with increasing BMI, but the trends differed between blacks and whites (p_interaction_ < .0001). Adjusted ORs (CIs) for diabetes among those with BMI≥40 vs 20–25 kg/m^2^ were 11.9 (8.4–16.8) for whites and 4.0 (3.3–4.8) for blacks. Diabetes incidence was more than twice as high among blacks than whites of normal BMI, but the racial difference became attenuated as BMI rose, with estimated 5-year probabilities of developing diabetes approaching 20% for both blacks and whites with BMI≥40 kg/m^2^. Diabetes risk was also associated with low SES, significantly (p_interaction_≤.02) more so for whites, current cigarette smoking, and lower healthy eating and physical activity indices, although high BMI remained the predominant risk factor among both blacks and whites. From baseline prevalence and 20-year projections of the incidence trends, we estimate that the large majority of surviving cohort participants with BMI≥40 kg/m^2^ will be diagnosed with diabetes.

**Conclusions:**

Even using conservative criteria to ascertain diabetes incidence (i.e., requiring diabetes medication use and ignoring undiagnosed cases), rates of obesity-associated diabetes were exceptionally high in this low-income adult population. The findings indicate that effective strategies to halt the rising prevalence of obesity are needed to avoid substantial increases in diabetes in coming years.

## Introduction

**Fig 1:**
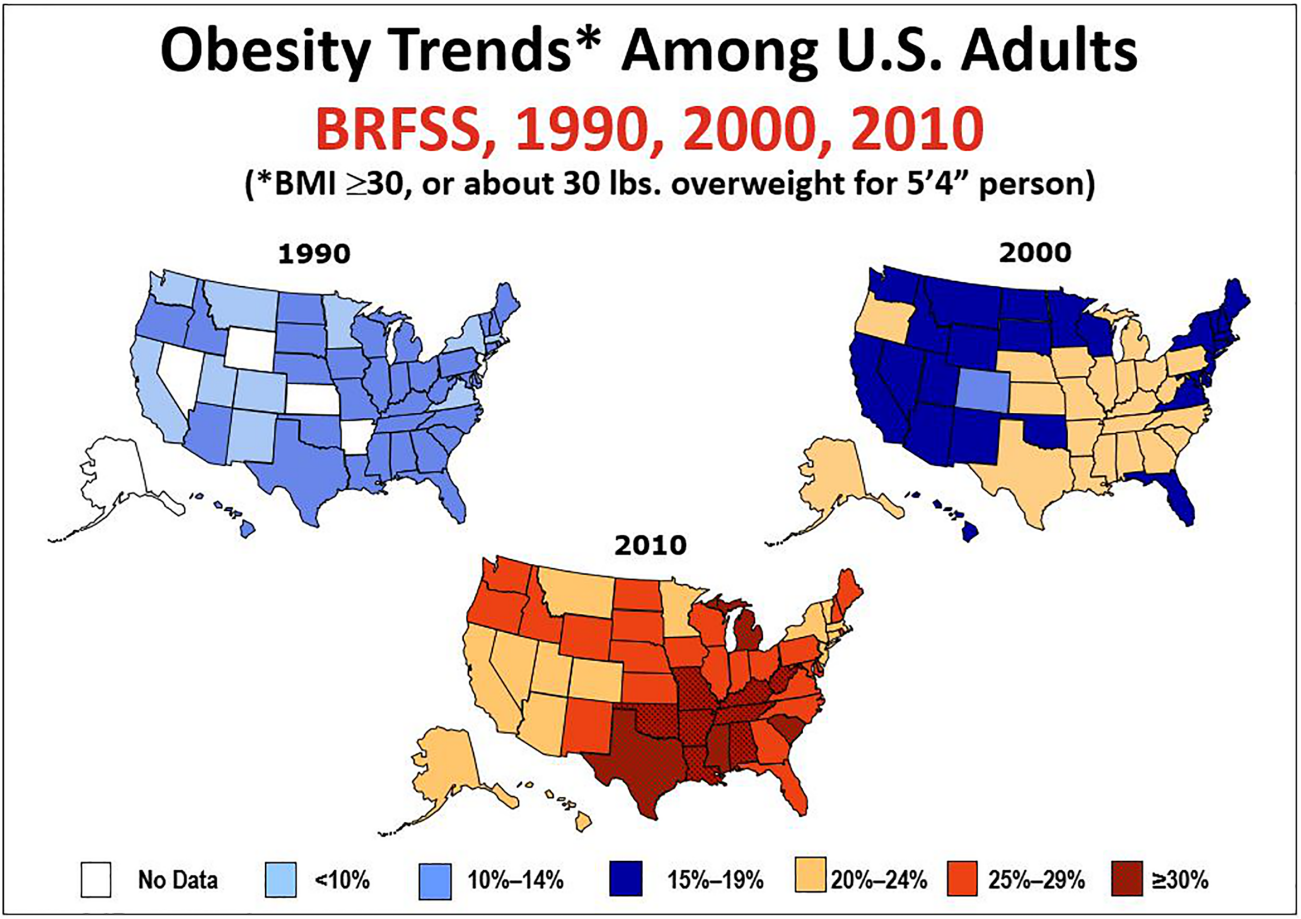
Source: Centers for Disease Control and Prevention, National Center for Chronic Disease Prevention and Health Promotion, Division of Population Health. BRFSS Prevalence & Trends Data: 1990, 2000, 2010.

National surveys have documented the rise in the prevalence of obesity in the United States since the 1980s [1–3]. Fig 1 shows the changes over time and the emerging geographic concentration of the highest rates of obesity occurring in the South. Obesity prevalence is also higher among blacks than whites and among groups of low income or education levels [1–4]. Obesity is known to be a major risk factor for Type 2 diabetes, with higher incidence among blacks than whites [5], but limited data exist on the magnitude of, and racial differences in, diabetes risk in southern US populations of low socioeconomic status where obesity prevalence is especially high.

**Fig 2:**
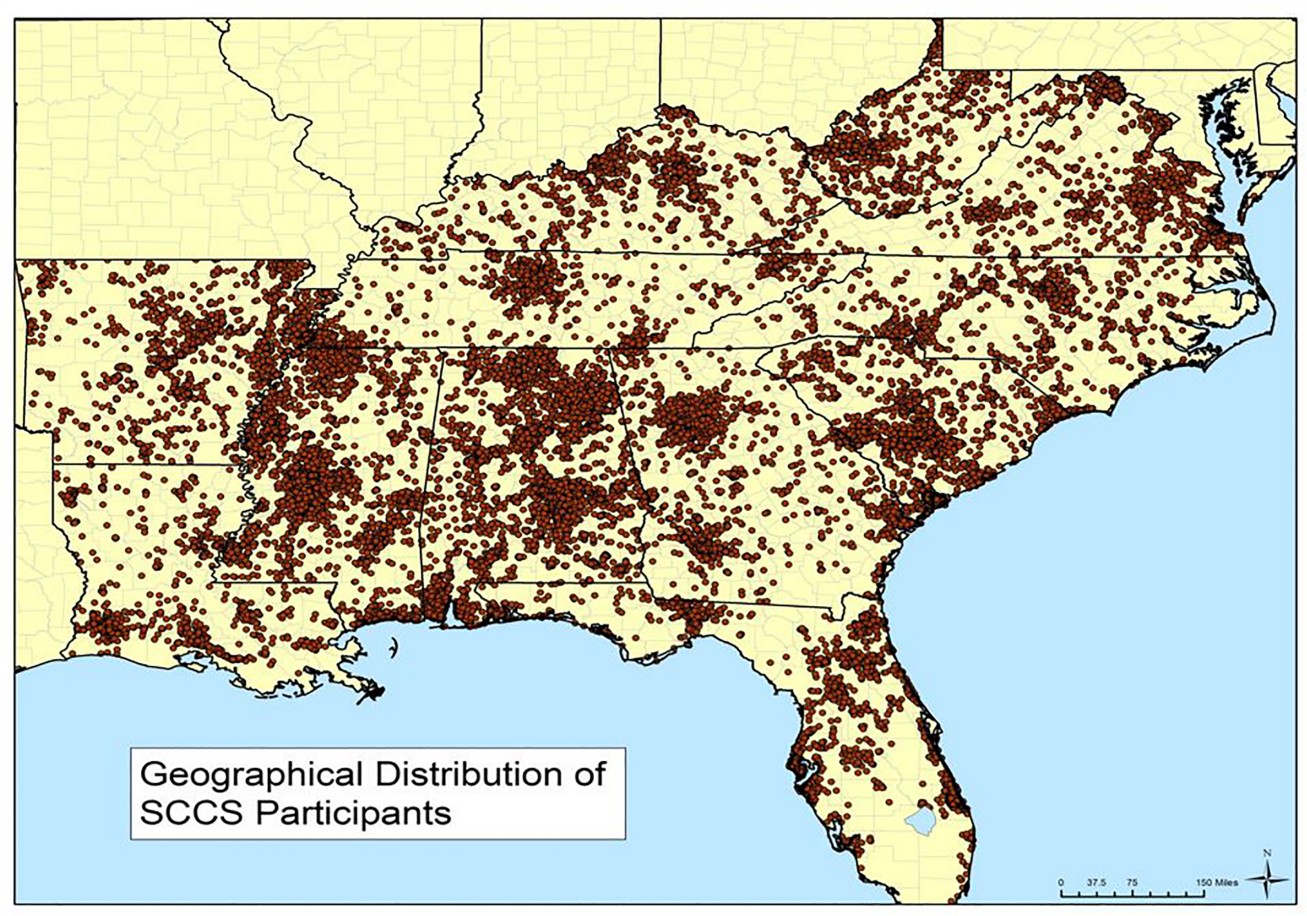
Geographical distribution of SCCS participants.

We are conducting prospective research within a diverse cohort of adults, many of low SES, who are residents across a broad southern area of the United States overlapping with the obesity belt (Fig 2). At cohort entry 44% of the participants were obese, with the obesity prevalence reaching 57% among black women [6]. The high prevalence of obesity may place the cohort at risk of future adverse health effects, particularly diabetes, a chronic illness rising in prevalence nationally [5]. Recent national statistics estimate that 12% of 45–64 year old Americans have diagnosed diabetes, with prevalence nearly twice as high among blacks than whites [5]. Herein we quantify rates of, and risk factors for, new-onset diabetes among blacks and whites in this large cohort. We focus on the association between obesity and diabetes, with the findings in this high-risk cohort providing clues to what may befall other American populations affected by the obesity epidemic.

## Methods

### Study design and participants

This research is carried out within the Southern Community Cohort Study (SCCS), an ongoing prospective cohort study investigating health disparities in diabetes, cancer, cardiovascular and other chronic diseases among African Americans and whites recruited mainly from underserved populations across a 12-state span (Alabama, Arkansas, Florida, Georgia, Kentucky, Louisiana, Mississippi, North Carolina, South Carolina, Tennessee, Virginia, and West Virginia). There were too few participants of racial groups other than black or white for meaningful statistical analysis. Details of study recruitment and follow up are provided elsewhere [6,7]. In brief, over 85,000 adults, two-thirds African American, between the ages of 40 and 79 were enrolled during 2002–2009. Approximately 85% were recruited in person at one of 71 community health centers (CHCs), with the remainder responding to mailings to stratified random samples of the general populations of these same states. The recruitment at CHCs, institutions providing basic health care and preventative services in underserved areas [8], resulted in a study population of generally low income and education levels for both blacks and whites. The enrollees completed a questionnaire, via personal interview at CHCs, with detailed information on demographic, socioeconomic, personal and family medical history, and lifestyle choices such as tobacco and alcohol use and diet. The cohort is followed for vital status via linkages with the National Death Index (NDI) and the Social Security Administration’s Service for Epidemiologic Research [9]. In addition, surviving participants are periodically sent follow-up questionnaires, on average about four years apart, to update selected information and inquire about health events, including diabetes. Copies of the baseline and follow-up questionnaires can be found on the study website www.southerncommunitystudy.org/questionnaires.html [7].

We recognize that the studied sample is not representative of the general population of the 12 states, not necessarily representative of all underserved residents, and may have had a higher baseline prevalence of diabetes because we recruited primarily from community health centers. However, the prevalences of both obesity and diabetes at cohort entry were also high among the 15% recruited from the general population. Although the SCCS cohort may not be a representative sample, validity of our results is not compromised, since within the selected population we collected information in the same way and applied the same assessments to all study subjects.

In the baseline questionnaire at cohort entry, respondents were asked, “Has a doctor ever told you that you have had, or have you been treated for, diabetes or high blood sugar (not during pregnancy)?” In the first and second follow-up questionnaires, respondents were asked, “After joining this study, have you been diagnosed with diabetes/high blood sugar?” Those answering “yes” were then asked, “Are you currently taking medication to control your diabetes?” For the study of incident diabetes herein, we restricted analyses to those cohort members who completed the first follow-up questionnaire and did not report diabetes at cohort entry. In our primary analyses we then defined incident diabetes as a report in the first follow-up questionnaire of diabetes with the taking of medicine to treat the diabetes. The restriction to those on anti-hyperglycemic medication was made to essentially eliminate the possibility of false positives among the cases. However, we also carried out secondary analyses using a self-report of diabetes, regardless of medication use, to define incident cases. We also carried out a replication analysis among those who did not report diabetes at either the baseline or first follow up, assessing incident diabetes with medication use diagnosed during the second follow-up period.

Current weight and height were self-reported in the baseline survey. For approximately 25% of the CHC participants, height and weight measured on the day of the baseline interview were abstracted from CHC medical records. The correlation between the self-reported and measured values of body mass index (BMI), calculated as [weight (kg)] / [height (m)^2^], was 0.96. BMI was categorized as <20, 20–24.9 (normal), 25–29.9 (overweight), and obesity classes I (30.0–34.9), II (35.0–39.9) and III (≥40), to enable comparisons with multiple prior reports using these standard categorizations [1–3,10–13], and also modeled as a continuous variable using cubic splines. Kappa values assessing concordance between BMI categories based on self report vs measurement were 0.80 (simple) and 0.88 (weighted), with 95% CI within +/- 1% of these values.

The SCCS was approved by the institutional review boards of Vanderbilt University and the Meharry Medical College and all participants gave written informed consent at study enrollment.

### Statistical analysis

We applied Pearson’s chi-square, analysis of variance (ANOVA), and Kruskal-Wallis tests to test for differences in study variables between blacks and whites. Because of uncertainty about the exact timing of onset of diabetes in the interval between cohort entry and administration of the first follow-up survey, rather than using Cox time-to-event modeling we employed logistic regression models to estimate ORs and 95% CIs for incident diabetes. We carried out the modeling separately for blacks and whites, and then combined with interaction tests to determine if the ORs associated with various participant characteristics differed significantly by race. Time between cohort entry and administration of the follow-up questionnaire (days) and the following demographic covariates were included in the models: age at cohort entry, sex, education, household income, recruitment source, and health insurance status. The key exposure variables of interest were BMI, cigarette smoking status, alcohol consumption, histories of hypertension and high cholesterol, a healthy eating index [14,15] composite score based on reported food intakes from the 89-item food frequency questionnaire, and an index of total physical activity based on estimation of ME-hours of energy expenditure associated with the various physical and sedentary activities queried [16]. Missing values tended to affect only a few percent of any covariate, so persons with any missing data were excluded from the logistic regression analyses. We carried out logistic regression modeling of BMI in traditional categories and as a continuous variable, using cubic B-splines with three knots placed at BMI 20, 30, and 40 kg/m^2^. We then graphed the relation between BMI and estimated 5-year probabilities of developing diabetes, attaining the estimates by setting the time between cohort entry and follow-up at 5 years and converting the predicted log-odds to probabilities. To estimate the long-term percentages of SCCS participants having diabetes in relation to obesity, we estimated 20-year diabetes cumulative incidence under the simplifying assumptions that the 5-year probabilities associated with BMIs of 30, 35 and 40 kg/m^2^ for SCCS participants without diabetes at cohort entry held for subsequent pentads and that there was no competing mortality. Age was not adjusted for in these projections, since in our logistic regression models age was not a significant predictor of diabetes incidence, with odds ratios of 0.99 (blacks) and 1.01 (whites) per increasing year of age. We then added these 20-year incidence estimates to the observed prevalences of diabetes among cohort participants at cohort entry at these BMI levels [17] to estimate the proportions of SCCS participants anticipated to have diabetes by the end of this 20-year span.

The primary logistic models compared those with incident diabetes reporting taking anti-hyperglycemic medication to those not reporting diabetes. Persons reporting diabetes but not taking medicines were excluded from the primary analysis. In sensitivity analyses, we classified all people self-reporting diabetes as cases and repeated the analyses we had done when diabetes cases were defined as those taking medications for diabetes, finding similar patterns (data not shown).

These same procedures were repeated for studying risk factors for medication-treated new-onset diabetes reported between the first and second SCCS follow-up surveys among the smaller number of participants who completed both follow-up surveys, with participants in this extended time analysis limited to those who did not report diabetes at baseline and at the first follow up. In this analysis, BMI was defined using weight at the time of the first follow up, whereas the other covariates were based on values reported at cohort entry. We report these analyses separately to provide risk estimates in this extended time period. In supplementary analyses, we combined the data from the first and second follow ups, finding generally similar results (data not shown).

All statistical tests were two-tailed with p-values <0.05 considered significant. Analyses were performed using SAS version 9.3 (SAS Institute Inc., Cary, NC, USA).

## Results

A total of 24,000 blacks and 14,064 whites who did not report diabetes at entry into the SCCS completed the first follow-up survey a median (and mean) of 4.5 years (range 1 to 10 years) after entry into the SCCS. Among blacks, 4,056 (17%) reported that they had been diagnosed with diabetes during the follow-up, with 2,671 (12%) reporting that they were taking anti-hyperglycemic medication for diabetes treatment. Among whites, 1,265 (9%) reported being newly diagnosed with diabetes, with 797 (6%) taking anti-hyperglycemic medication. Table 1 shows demographic characteristics of those with incident diabetes (defined as reporting diabetes and taking anti-hyperglycemic medication) and of those not reporting diabetes. Differences between the diabetes cases and non-cases by age and sex were small among both blacks and whites, but the incident cases were more likely to be of lower education and income status and less likely to have private or other non-Medicaid/Medicare health insurance than those without diabetes, with the differences more pronounced among whites than blacks.

**Table 1:** Demographic characteristics of study participants by diabetes status among blacks and whites.

	Black cases^a^	Black non-cases	White cases^a^	White non-cases
	n = 2,671	n = 19,944	n = 797	n = 12,799
**Mean age at enrollment (yrs)**	51.5	51.3	54.5	54.5
**Sex**				
Male	34.3%	35.0%	35.4%	36.3%
Female	65.7%	65.0%	64.6%	63.7%
**Education**				
< High school	32.3%	24.4%	30.4%	16.4%
High school/vocational	39.8%	39.4%	36.7%	35.1%
> = High school	27.9%	36.1%	32.8%	48.5%
**Income**				
< $15,000	56.8%	52.4%	49.3%	35.0%
$15,000-$24,999	24.3%	23.2%	19.9%	17.1%
> = $25,000	18.9%	24.4%	30.8%	47.9%
**Insurance coverage**				
No insurance	38.3%	39.1%	38.4%	28.9%
Medicaid/Medicare	35.1%	30.3%	33.3%	29.4%
Other	26.6%	30.6%	28.4%	41.7%

^a^ Cases are defined as those who reported ‘YES’ for incident diagnoses of diabetes and ‘YES’ for medications of diabetes at follow up 1; Non-cases are those who reported ‘NO’ for diabetes diagnoses.

The percentage of individuals diagnosed with diabetes did not vary greatly between men and women, but rose sharply with increasing BMI (Table 2). Nearly 20% of blacks and 17% of whites with class III obesity (BMI≥40 kg/m^2^) reported taking medicines to treat new-onset diabetes between cohort entry and first follow up, whereas the corresponding figures among those with BMI<25 kg/m^2^ were less than 6% among blacks and less than 2% among whites. Table 2 also shows that unadjusted diabetes incidence was higher among white smokers, but not black smokers, lower among drinkers of alcoholic beverages, and modestly lower among those with healthy eating and higher physical activity index scores. Diabetes onset was also increased among both blacks and whites with histories of elevated cholesterol or hypertension.

**Table 2:** Cumulative diabetes incidence (%) during follow up according to sex, BMI, tobacco and alcohol consumption, comorbidity, and healthy eating and physical activity indices among blacks and whites.

	Blacks		Whites	
	N	% with diabetes^a^	N	% with diabetes^a^
**Sex**				
Male	7,891	11.6	4,927	5.7
Female	14,724	11.9	8,669	5.9
**BMI (kg/m**^**2**^**)**				
< 20	726	5.6	550	0.7
20–24.9	4,539	5.9	3,551	1.6
25–29.9	6,884	10.2	4,508	4.2
30–34.9	5,083	13.5	2,758	7.7
35–39.9	2,743	17.3	1,169	14.3
> = 40	2,327	19.6	916	16.8
**Smoking status**				
Current	8,715	11.4	4,120	6.8
Former	4,574	13.3	4,148	6.0
Never	9,146	11.4	5,189	5.1
**Alcohol consumption**				
Non-drinker	9,899	13.1	6,135	7.5
< 1 per day	7,317	11.1	4,960	4.9
> = 1 per day	5,039	10.2	2,240	3.5
**High cholesterol**				
Yes, with statin	2,436	15.0	2,586	8.5
Yes, otherwise	3,678	13.7	2,743	6.3
No	16,461	10.9	8,249	4.9
**Hypertension**				
Yes	12,074	15.2	5,765	9.0
No	10,533	7.9	7,826	3.5
**Healthy eating index**				
Q1	5,138	12.2	3,407	6.7
Q2	5,509	12.5	3,023	6.4
Q3	5,410	11.8	3,108	6.3
Q4	5,032	10.7	3,584	4.1
**Total activity MET-hrs**				
Q1	5,433	13.8	3,307	7.6
Q2	5,417	11.7	3,398	5.6
Q3	5,448	11.3	3,417	5.3
Q4	5,732	10.2	3,045	4.8

^a^ Cases are defined as those who reported ‘YES’ for incident diagnoses of diabetes and ‘YES’ for medications of diabetes at follow up 1.

In multivariate adjusted logistic regression models, BMI persisted as the dominant diabetes risk factor among both blacks and whites, but the adjustments revealed about 25% lower diabetes risks among women than men (Table 3). Interaction tests showed that the association between diabetes risk and BMI differed significantly (p < .0001) between blacks and whites, with stronger trends seen among whites. Nearly 10-fold or greater increases in risk among those with BMI≥35 kg/m^2^ vs normal weight were observed among whites vs 3-to-4-fold increases among blacks. The links between diabetes and low SES, particularly lack of a high school diploma, were also more pronounced among whites than blacks. Cigarette smoking was associated with an up to 40% increased diabetes risk, while alcohol consumption was associated with reduced risk. Those reporting hypertension and high cholesterol were also more likely to be diagnosed with diabetes, with the excess greater among those using statins to control their hyperlipidemia. Inverse associations between diabetes onset and healthier eating and higher levels of physical activity were seen, but the protective trends were modest and significant only for physical activity. In sensitivity analyses classifying all people self-reporting diabetes as cases, ORs were nearly the same as shown in Table 3, although somewhat attenuated in the total case vs medication-only case analyses.

**Table 3:** Adjusted odd ratios for incident diabetes according to sex, BMI, tobacco and alcohol consumption, comorbidity, and healthy eating and physical activity indices among blacks and whites.

	Black		White		
	OR (95% CI)	p-value	OR (95% CI)	p-value	p-interaction^a^
**Sex**					
Female vs male	0.74 (0.67–0.82)	<0.0001	0.75 (0.62–0.90)	0.002	0.02
**BMI**		<0.0001		<0.0001	<0.0001
< 25	Ref		Ref		
25–29.9	1.76 (1.51–2.05)		2.74 (1.99–3.76)		
30–34.9	2.43 (2.07–2.84)		4.95 (3.60–6.79)		
35–35.9	3.25 (2.74–3.87)		9.10 (6.50–12.73)		
> = 40	3.97 (3.33–4.75)		11.87 (8.38–16.82)		
**Education**		<0.0001		<0.0001	0.02
< High school	Ref		Ref		
High school/vocational	0.82 (0.74–0.92)		0.73 (0.60–0.90)		
> = High school	0.67 (0.59–0.77)		0.57 (0.45–0.72)		
**Income**		0.13		0.11	0.02
< $15,000	Ref		Ref		
$15,000-$24,999	1.04 (0.93–1.16)		0.92 (0.74–1.14)		
> = $25,000	0.89 (0.78–1.03)		0.77 (0.60–0.98)		
**Insurance coverage**		0.67		0.02	0.02
No insurance	Ref		Ref		
Medicaid/Medicare	1.03 (0.92–1.15)		0.74 (0.61–0.91)		
Other	0.97 (0.86–1.10)		0.84 (0.66–1.06)		
**Smoking status**		0.004		0.0006	0.55
Never	Ref		Ref		
Former	1.13 (1.00–1.27)		1.03 (0.84–1.26)		
Current	1.21 (1.08–1.35)		1.46 (1.18–1.80)		
**Alcohol consumption**		0.10		0.01	0.01
Non-drinker	Ref		Ref		
<1 per day	0.95 (0.85–1.05)		0.88 (0.73–1.05)		
≥1 per day	0.87 (0.76–0.99)		0.66 (0.50–0.87)		
**High cholesterol**		0.006		<0.0001	0.11
No	Ref		Ref		
Yes, with statin	1.22 (1.06–1.40)		1.55 (1.27–1.90)		
Yes, otherwise	1.14 (1.02–1.29)		1.13 (0.92–1.40)		
**Hypertension**					
Yes vs No	1.90 (1.72–2.10)	<0.0001	1.65 (1.38–1.96)	<0.0001	0.54
**Healthy eating index (HEI10)**		0.37		0.78	0.81
Q1	Ref		Ref		
Q2	1.06 (0.93–1.20)		1.00 (0.81–1.24)		
Q3	1.00 (0.88–1.14)		1.08 (0.86–1.35)		
Q4	0.94 (0.82–1.08)		0.95 (0.74–1.22)		
**TotalActivityMetHr**		0.03		0.35	0.89
Q1	Ref		Ref		
Q2	0.88 (0.78–0.99)		0.87 (0.70–1.08)		
Q3	0.89 (0.78–1.01)		0.90 (0.72–1.12)		
Q4	0.83 (0.73–0.94)		0.81 (0.64–1.03)		

^a^ Interaction with race, tested individually for each covariate.

**Fig 3:**
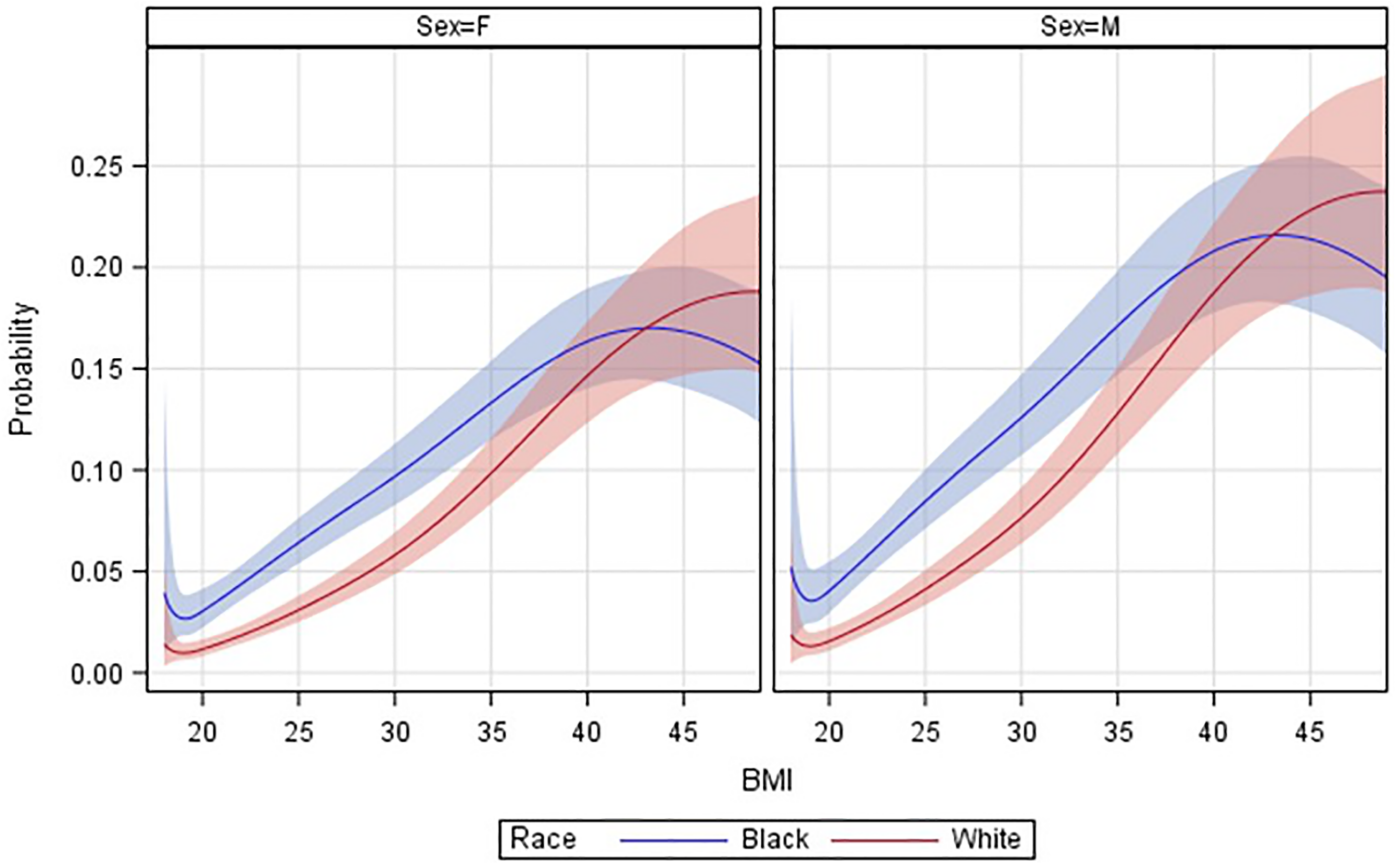
Plots of estimated 5-year probabilities of incident medication-treated diabetes during mean follow-up by BMI according to race and sex^1^. ^1^ Estimated 5-year probabilities of incident diabetes specific for a person who was enrolled at mean values of enrollment age, alcohol drinks per day and total physical activity MET-hours, and modal values for other categorical covariates, with cubic spline knots at BMI 20, 30 and 40 kg/m^2^; Shaded bands about the curves represent 95% confidence limits on the estimated probabilities.

Fig 3 presents graphs of predicted 5-year probabilities of incident diabetes by sex and race according to BMI modeled using cubic splines and with covariates in the multivariate models set at mean or modal levels. Among both men and women the estimated probabilities of diabetes rise with BMI, but the black excess apparent among the non-obese becomes attenuated as BMI rises above 35 kg/m^2^ and disappears at the highest BMI levels.

Table 4 shows the percentages of SCCS surviving participants projected to have diabetes by the end of the next two decades. The percentages rose steadily with increasing BMI, with over two-thirds (and three-fourths of men), regardless of race, with BMIs of 40 kg/m^2^ affected.

**Table 4:** Projected approximate percentages^1^ of participants with diabetes 20 years after entry into the SCCS by sex, race for selected BMI levels.

		BMI (kg/m^2^)		
Sex	Race	30	35	40
Female	White	32%	49%	65%
	Black	46%	58%	68%
Male	White	37%	58%	76%
	Black	51%	66%	75%

^1^ Sum of baseline prevalence at cohort entry plus estimated 20-year probabilities of incident diabetes specific for a person who was enrolled at mean values of enrollment age, alcohol drinks per day and total physical activity MET-hours, and modal values for other categorical covariates. 20-year estimates based on extrapolation of 5-year cubic spline estimates assuming 5-year estimates persisted and assuming no competing risks from mortality.

A total of 12,834 blacks and 9,718 whites who did not report diabetes in either the baseline or first follow-up survey completed the second follow-up survey a median of 3 (range ≤1 to 7) years after the first follow-up survey. In analyses in this subset, findings were nearly the same as those seen in assessing incident diabetes between cohort entry and the first follow-up survey. Table 5 lists the percentages newly reporting incident diabetes at the second follow up, with Table 6 showing corresponding ORs of diabetes associated with BMI and the other variables examined. In brief, incident medication-treated diabetes was reported by 11% of blacks and 5% of whites, with the percentages rising to 20% among blacks and 16% among whites with BMI≥40 kg/m^2^. Adjusted diabetes ORs were 2.8, 5.3, 6.3 and 12.1 among whites respectively with BMI 25–29, 30–34, 35–39 and ≥40 relative to 20–24 kg/m^2^ and 1.6, 2.1, 3.3 and 3.7 among blacks, a highly significant difference (p_interaction_ < .0001). Associations with SES, smoking and drinking, and comorbidities also were generally similar to those in the first follow up, although the lower ORs associated with high physical activity were not seen, with lower ORs now appearing among those with high healthy eating index scores.

**Table 5:** Cumulative diabetes incidence (%) during second follow up according to sex, BMI, tobacco and alcohol consumption, comorbidity, and healthy eating and physical activity indices among blacks and whites.

	Blacks		Whites	
	N	% with diabetes^a^	N	% with diabetes^a^
**Sex**				
Male	3,795	10.1	3,353	5.0
Female	7,799	11.4	5,942	4.8
**BMI (kg/m**^**2**^**)**				
< 20	365	5.2	389	1.0
20–24.9	2,176	5.6	2,482	1.3
25–29.9	3,515	9.4	3,162	3.8
30–34.9	2,659	11.7	1,787	7.4
35–39.9	1,385	17.2	757	9.2
> = 40	1,117	19.7	537	15.8
**Smoker at FU1**				
No	7,777	10.7	7,073	4.8
Yes	3,700	11.5	2,162	5.1
**Alcohol consumption**				
Non-drinker	5,071	11.4	3,954	6.0
< 1 per day	3,851	10.9	3,581	4.4
> = 1 per day	2,479	10.2	1,584	3.5
**High cholesterol**				
Yes, with statin	1,290	13.3	1,776	6.7
Yes, otherwise	1,900	12.5	1,871	5.2
No	8,387	10.3	5,642	4.2
**Hypertension**				
Yes	5,801	14.2	3,630	7.6
No	5,790	7.7	5,661	3.2
**Healthy eating index**				
Q1	2,659	11.5	2,247	6.3
Q2	2,860	12.0	2,083	5.8
Q3	2,736	10.7	2,204	4.7
Q4	2,585	9.6	2,478	2.9
**Total activity MET-hrs**				
Q1	2,777	9.9	2,235	4.7
Q2	2,746	10.6	2,326	5.2
Q3	2,726	11.1	2,345	4.6
Q4	3,001	12.0	2,075	5.1

^a^ Cases are defined as those who reported ‘YES’ for incident diagnoses of diabetes and ‘YES’ for medications of diabetes at Follow Up 2. All participants reported not having diabetes at baseline and at Follow Up 1.

**Table 6:** Adjusted odd ratios for incident diabetes during the second follow up according to sex, BMI, tobacco and alcohol consumption, comorbidity, and healthy eating and physical activity indices among blacks and whites.

	Black		White		
	OR (95% CI)	p-value	OR (95% CI)	p-value	p-interaction^a^
**Sex**					
Female vs male	0.88 (0.76–1.03)	0.11	0.72 (0.57–0.91)	0.007	0.86
**BMI**		<0.0001		<0.0001	<0.0001
< 25	Ref		Ref		
25–29.9	1.62 (1.30–2.02)		2.84 (1.88–4.27)		
30–34.9	2.08 (1.66–2.61)		5.25 (3.49–7.90)		
35–35.9	3.28 (2.57–4.19)		6.25 (3.98–9.83)		
> = 40	3.68 (2.84–4.76)		12.08 (7.71–18.93)		
**Education**		0.14		0.005	0.003
< High school	Ref		Ref		
High school/vocational	0.99 (0.83–1.18)		0.71 (0.53–0.94)		
> = High school	0.86 (0.71–1.04)		0.60 (0.43–0.82)		
**Income**		0.13		0.04	0.02
< $15,000	Ref		Ref		
$15,000-$24,999	1.12 (0.95–1.32)		0.98 (0.74–1.31)		
> = $25,000	0.92 (0.77–1.12)		0.69 (0.51–0.93)		
**Insurance coverage**		0.42		0.72	0.38
No insurance	Ref		Ref		
Medicaid/Medicare	1.02 (0.86–1.21)		1.07 (0.79–1.44)		
Other	0.91 (0.76–1.09)		0.95 (0.68–1.31)		
**Smoker**		0.003		0.79	0.05
Yes vs No	1.27 (1.09–1.48)		0.96 (0.74–1.26)		
**Alcohol consumption**		0.85		0.40	0.04
Non-drinker	Ref		Ref		
<1 per day	1.05 (0.90–1.21)		0.98 (0.78–1.24)		
≥1 per day	1.02 (0.84–1.23)		0.75 (0.56–1.12)		
**High cholesterol**		0.27		0.03	0.41
No	Ref		Ref		
Yes, with statin	1.16 (0.94–1.42)		1.43 (1.10–1.87)		
Yes, otherwise	1.10 (0.93–1.31)		1.13 (0.87–1.48)		
**Hypertension**		<0.0001		<0.0001	0.33
Yes vs no	1.76 (1.53–2.03)		1.62 (1.29–2.02)		
**Healthy eating index (HEI10)**		0.08		0.12	0.43
Q1	Ref		Ref		
Q2	1.09 (0.91–1.29)		0.98 (0.75–1.29)		
Q3	0.91 (0.75–1.09)		0.87 (0.65–1.16)		
Q4	0.86 (0.71–1.06)		0.69 (0.49–0.96)		
**TotalActivityMetHr**		0.009		0.04	0.28
Q1	Ref		Ref		
Q2	1.07 (0.88–1.29)		1.52 (1.13–2.05)		
Q3	1.27 (1.05–1.54)		1.39 (1.02–1.89)		
Q4	1.32 (1.09–1.59)		1.43 (1.04–1.96)		

^a^Interaction with race, tested individually for each covariate.

## Discussion

The analyses in this low-income southern adult population indicate that the obesity epidemic in the United States, most prominent in the South and among groups with lower levels of education and income, has led to marked increases in the incidence and prevalence of diabetes. Over only a median 4.5-year follow-up period, nearly 12% of blacks and 6% of whites overall, and 20% and 17% who were morbidly obese (BMI≥40 kg/m^2^), reported developing adult-onset diabetes requiring medication treatment. These high incidence rates were confirmed in the extended analyses adding another median 3 years of follow up. On top of an already high prevalence of diabetes at cohort entry (approximately 20% overall, ranging upwards to 40% among those with BMI≥40 kg/m^2^) [17], with the elevated incidence described in this current report, we can project that diabetes within two decades will be diagnosed in the majority of obese (BMI≥30 kg/m^2^) SCCS participants, and approximately 75% of morbidly obese (BMI≥40 kg/m^2^) men. These high percentages were estimated using a conservative definition of diabetes, requiring use of medications to treat the condition and ignoring undiagnosed cases. Nationally, diabetes prevalence has been rising, although overall prevalence (diagnosed plus undiagnosed) among adults aged 45–64 reached only 18% [6]. Our striking findings suggest that the SCCS cohort may be a harbinger signaling further increases to come in other American population components where obesity is common.

High BMI was the dominant risk factor for diabetes among both whites and blacks, but we found significantly stronger associations between BMI and incident diabetes among whites. The higher ORs for the obesity class I, II and III categories, respectively 30–34, 35–39 and ≥40 kg/m^2^, among whites are in part reflective of considerably lower absolute diabetes risk among whites than blacks for those of normal weight, the reference category for the OR calculations. This lower absolute risk of diabetes among whites than blacks in the normal weight and overweight/non-obese range has also been reported by others [18–20]. Our findings indicate, however, that the black excess became attenuated as BMI rose, and at very high obesity levels diabetes became so common, regardless of race, that the racial differences tended to disappear.

Although the SES of the SCCS population is lower than in most other cohorts, there was sufficient variation to demonstrate that diabetes incidence tended to decline as both education and income levels rose. Risk of incident diabetes was more closely related to education than income, being 33% to 45% lower among those with beyond vs less than high school educations even after adjusting for BMI, income, health insurance status and other risk factors for the illness. The excess risk among the most disadvantaged may be a signal of social determinants of diabetes, and suggests that obesity and diabetes prevention messages targeted to groups with less education may be beneficial.

We have previously reported that the percentages of SCCS participants who smoked cigarettes markedly declined with rising BMI [21]. Despite this inverse association, we found smoking to be associated with significantly increased risk of diabetes, with about a 25% excess among current smokers. Our large study size and tight control for BMI and other predictors of diabetes risk helped enable the detection of the smoking effect. Early (1964–2004) reports of the Surgeon General on smoking did not list diabetes as a smoking inducible disease [22–24], but meta analyses in the 2014 Surgeon General’s report on 50 years of progress in smoking research noted a 30% to 40% increased risk of type 2 diabetes among smokers [25]. Smoking thus can be considered a modifiable cause of diabetes, but smoking cessation strategies aimed at diabetes reduction need to incorporate weight control components since quitting smoking has been associated with weight gain [26–29]. While smoking and drinking behaviors tend to be correlated, we found decreased rather than increased risks of diabetes among daily alcohol drinkers, with the association stronger among whites than blacks. Others have also reported a reduced risk of type 2 diabetes among moderate alcohol drinkers, with some indication of a U-shaped relationship with risk being elevated among heavy drinkers [30–32]. Levels of alcohol consumption were relatively low among SCCS participants, with only about 20% of cohort members reporting daily drinking, so that our ability to detect an upturn in risk at high levels of intake was limited.

We also examined other potential modifiable causes of diabetes, including broad markers of physical activity and diet. The SCCS baseline questionnaire ascertained information on recreational and occupational physical activity, as well as sedentary activity, and included a detailed food frequency questionnaire tailored to the Southern diet, from which we derived summary indices of physical activity and “healthy eating” [33–35]. In unadjusted analyses, both were linked with moderately lower diabetes incidence. After adjustment for BMI and other risk factors, the associations were attenuated, but significantly lower diabetes risk persisted among those with higher levels of physical activity, although this was one of the few associations not confirmed in our extended follow-up analysis. The modest (rather than strong) associations of physical activity and diet with diabetes observed in our study could arise from our inability to accurately classify these lifestyle activities, but also could be indicative of overall lack of adequate physical activity and healthy diets among underserved groups. Indeed, we have previously reported that less than one-fourth of SCCS participants adhered to recommended national physical activity guidelines [36]. The findings suggest that diet and physical activity alone, at least within the range experienced by SCCS participants, may not be sufficient to overcome the strong adverse effects of obesity on diabetes incidence.

We examined associations of incident diabetes with histories of hypertension and hyperlipidemia reported at entry into the cohort. As expected, diabetes onset was much more frequent among those with hypertension, and increased to a lesser extent among those with high cholesterol, with the effect greater among those taking statins. The elevation in diabetes risk was relatively small (13%-14%) among persons with unmedicated high cholesterol, but among whites rose to 55% (95% CI 27%-90%) among those treated with statins. Although it is difficult for observational studies to sort out effects of drugs from effects of the conditions requiring treatment by the drugs, our findings raise the possibility of an impact of dyslipidemia itself with an additional impact of statins. We had no information on indications for use, and could not discern whether statin users might have had more severe hyperlipidemia than non-statin users. However, several lines of evidence suggest an adverse impact of statins on diabetes: statins have been reported to influence insulin sensitivity; increased diabetes risk associated with statin use has been frequently reported; and Mendelian randomization studies show elevated diabetes risk among persons with genetic allelic variants associated with LDL cholesterol lowering [37–39]. Few of these studies, however, have included large numbers of blacks.

The large size of the cohort being followed and its unique composition of underserved individuals, both black and white, are study strengths since these high-risk groups are often not included in sizeable numbers in health studies. The systematic ascertainment of detailed data on multiple potential diabetes risk factors also adds to study strengths. Limitations include our reliance on self-report of diabetes, which prompted our further restriction of defining incident cases as those who also reported that they were taking medication to treat their diabetes. While this is a common practice [5], some misclassification could occur. In prior SCCS validation studies, self-report of diabetes was found to be highly specific [40]. We recognize that diabetes may be undiagnosed or unreported in a fraction of the population, so that some non-cases are misclassified. Indeed, in a recent sample of adult Americans from the National Health and Nutrition Examination Survey, over a third of all diabetes was thought to be undiagnosed [5]. In random samples of SCCS participants among whom A1C was measured as part of a panel of biomarkers for two prior studies, among those not reporting diabetes at baseline or at the follow-up survey, 4% of whites but 10%-20% of blacks had baseline A1C levels of 6.5% or higher. Hence, more of the non-cases among blacks may have had undetected diabetes; the resulting misclassification would be expected to weaken the ORs more for blacks than whites and could contribute in part to the black-white differences we observed in associations between BMI, SES and diabetes. Since misclassification tends to lower ORs, the strong associations reported herein among both whites and blacks may actually be underestimated. Further, the already high diabetes incidence rates may also be underestimates of the diabetes burden in this at-risk cohort. We note that the high prevalence of diabetes at baseline may in part have been due to our recruitment from community health centers, and that the high starting point contributes to our projections that diabetes will affect the large majority of morbidly obese SCCS participants in the next two decades.

Diabetes not only adversely affects quality of life, but can lead to serious and life-threatening complications, including blindness, leg amputation, and heart, kidney and other diseases [41–50]. We have previously reported that overall mortality rates are nearly twice as high among SCCS participants who did vs did not report diabetes at cohort entry [48], with over 2-fold increases in cardiovascular disease and 4-fold increases in renal disease deaths [49]. As shown herein, the major risk factor for this illness within the SCCS is obesity, a condition that is modifiable and preventable. Improved approaches to tackle the obesity problem need be developed and implemented. The striking findings within the SCCS highlight the urgency of the problem in an underserved population, and provide an additional wake-up call regarding potential future implications of the national obesity epidemic.
